# Structure of the protein core of translation initiation factor 2 in apo, GTP-bound and GDP-bound forms

**DOI:** 10.1107/S0907444913006422

**Published:** 2013-04-23

**Authors:** Angelita Simonetti, Stefano Marzi, Attilio Fabbretti, Isabelle Hazemann, Lasse Jenner, Alexandre Urzhumtsev, Claudio O. Gualerzi, Bruno P. Klaholz

**Affiliations:** a Department of Integrated Structural Biology, IGBMC (Institute of Genetics and of Molecular and Cellular Biology), Centre National de la Recherche Scientifique (CNRS) UMR 7104/Institut National de la Santé de la Recherche Médicale (INSERM) U964/Université de Strasbourg, 1 Rue Laurent Fries, 67404 Illkirch, France; b Architecture et Réactivité de l’ARN, UPR 9002 CNRS, IBMC (Institute of Molecular and Cellular Biology), 15 Rue R. Descartes, 67084 Strasbourg, France; Université de Strasbourg, 67000 Strasbourg, France; c Laboratory of Genetics, Department of Biosciences and Biotechnology, University of Camerino, 62032 Camerino (MC), Italy; d Physical Department, Université de Lorraine, 54506 Vandoeuvre-lès-Nancy, France

**Keywords:** translation initiation factor 2, *Thermus thermophilus*, GTP, GDP

## Abstract

Translation initiation factor 2 (IF2) is involved in the early steps of bacterial protein synthesis. It promotes the stabilization of the initiator tRNA on the 30S initiation complex (IC) and triggers GTP hydrolysis upon ribosomal subunit joining. While the structure of an archaeal homologue (a/eIF5B) is known, there are significant sequence and functional differences in eubacterial IF2, while the trimeric eukaryotic IF2 is completely unrelated. Here, the crystal structure of the apo IF2 protein core from *Thermus thermophilus* has been determined by MAD phasing and the structures of GTP and GDP complexes were also obtained. The IF2–GTP complex was trapped by soaking with GTP in the cryoprotectant. The structures revealed conformational changes of the protein upon nucleotide binding, in particular in the P-loop region, which extend to the functionally relevant switch II region. The latter carries a catalytically important and conserved histidine residue which is observed in different conformations in the GTP and GDP complexes. Overall, this work provides the first crystal structure of a eubacterial IF2 and suggests that activation of GTP hydrolysis may occur by a conformational repositioning of the histidine residue.

## Abstract 2

The crystal structures of the eubacterial translation initiation factor 2 in apo form and with bound GDP and GTP reveal conformational changes upon nucleotide binding and hydrolysis, notably of the catalytically important histidine in the switch II region.

## 1. Introduction

Protein synthesis in bacteria involves about ten distinct translation factors that assure processivity and accuracy of translation. They form transient complexes with the ribosome and enable the steps of translation initiation, elongation, termination and recycling to proceed correctly. Four of these factors are ribosomal GTPases (Bourne *et al.*, 1991 ▶): elongation factors Tu (EF-Tu) and G (EF-G), release factor 3 (RF3) and initiation factor 2 (IF2). In contrast to the other ribosomal GTPases, which exclusively bind to the 70S ribosome, IF2 primarily binds to the 30S subunit. During formation of the 30S initiation complex (30S IC), IF2 in its GTP-bound form stabilizes initiator fMet-tRNA^fMet^ on the small ribosomal subunit by interacting with the tRNA CCA acceptor end (Guenneugues *et al.*, 2000 ▶), which results in cooperative binding of the tRNA and IF2 on the 30S subunit (Simonetti *et al.*, 2008 ▶). Interestingly, the fMet-tRNA^fMet^ adopts an intermediary P/I (possibly high-energy) state which is stabilized by IF2–GTP (Simonetti *et al.*, 2008 ▶); consistently, this active state of IF2 is promoted by both the GTP molecule and the fMet-tRNA^fMet^ (Pavlov *et al.*, 2011 ▶). IF2 is also responsible for the fast subunit association and 70S IC formation, an event that is directly coupled to the activation of the IF2 GTPase (Tomsic *et al.*, 2000 ▶; Antoun *et al.*, 2006 ▶; Grigoriadou *et al.*, 2007 ▶; Marshall *et al.*, 2009 ▶; Tsai *et al.*, 2012 ▶). While the crystal structure of an archaeal homologue of IF2, a/eIF5B, is known (Roll-Mecak *et al.*, 2000 ▶), it has significant differences in sequence and function; for example, it plays no role in binding fMet-tRNA^fMet^ to the ribosome (Tomsic *et al.*, 2000 ▶; Antoun *et al.*, 2006 ▶; Grigoriadou *et al.*, 2007 ▶; Tsai *et al.*, 2012 ▶) and it primarily serves to favour ribosomal subunit joining (Pestova *et al.*, 2000 ▶). Furthermore, in the a/eIF5B structures (free, with GDP or with GDPNP) some functionally important regions were not always visible. Here, we present the crystal structure of the eubacterial IF2 core as the individual apoprotein and in GTP-bound and GDP-bound forms. The structures provide details of the molecular mechanism of nucleotide binding and suggest that GTP hydrolysis is activated by conformational change of a conserved histidine residue.

## 2. Experimental procedures

### 2.1. Protein preparation and crystallization

The expression and purification of full-length *Thermus thermophilus* IF2 (residues 1–571) without an affinity tag were performed as described in Thompson & Dahlberg (2004 ▶) except that additional hydrophobic chromatography steps were performed on Phenyl Sepharose and BTP650 columns (Simonetti *et al.*, 2008 ▶). The final buffer was 20 m*M* HEPES pH 7.5, 50 m*M* KCl, 20 m*M* MgCl_2_, 1 m*M* DTT. The integrity of IF2 was confirmed by gel electrophoresis followed by MALDI peptide mass fingerprinting analysis. The purified IF2 was characterized using dynamic light scattering (DynaPro DLS system) to evaluate the solution properties and the aggregation state of the protein. The DLS experiment was conducted at an IF2 concentration of 4 mg ml^−1^ (as also used in crystallization trials) and revealed a single peak with only 15% polydispersity, indicating an essentially monodisperse sample with no aggregation. Crystals of the 1–363 fragment (proteolytic cleavage site similar to that described by Szkaradkiewicz *et al.*, 2000 ▶; see Supplementary Material^1^ for characterization) suitable for X-ray analysis were grown at 294 K *via* sitting-drop vapour diffusion by mixing 8 µl reservoir solution consisting of 20% PEG 3350 and 0.2 *M* ammonium nitrate as precipitants with the same volume of protein solution (in 20 m*M* HEPES pH 7.5, 50 m*M* KCl, 20 m*M* MgCl_2_, 1 m*M* DTT); the protein solution was supplemented with 2.5% glycerol beforehand, incubated for 30 min at 310 K and kept at 277 K overnight. Crystals grew within 1–3 weeks to dimensions of up to ∼130 × 130 × 260 µm (the crystals of the GTP complex were smaller). For phasing, selenomethionine-labelled protein was prepared using the methionine-auxotroph *Escherichia coli* strain B834 (DE3) grown in a medium in which methionine was substituted by selenomithionine (SeMet). The IF2 purification protocol was identical to that for native IF2. The molecular weight of SeMet IF2 as well as the degree of substitution by SeMet was determined by electrospray ionization mass spectrometry (ESI-MS^+^), confirming the presence of all 15 methionines in IF2. Single crystals were grown using the same crystallization condition as used for the native protein, with a protein concentration of 5 mg ml^−1^. Crystal soaking with nucleotides was performed by adding 5 m*M* of GDP directly to the crystallization drop or by a 30 s incubation with GTP-containing cryoprotectant buffer. The occupancy of the GTP refined to 0.68, indicating that the GTP may have hydrolyzed partially in solution (IF2 has very low GTPase activity in the absence of the ribosome; however, the fast crystal soaking and cooling allowed the unstable GTP, which tends to hydrolyze spontaneously in solution, to be trapped). Crystals were cryoprotected with reservoir solution supplemented with 15% glycerol and flash-cooled in liquid nitrogen. The crystals belonged to the orthorhombic space group *P*2_1_2_1_2_1_ with one molecule in the asymmetric unit (Table 1 ▶).

### 2.2. Data collection, structure determination and refinement

**Figure 1:**
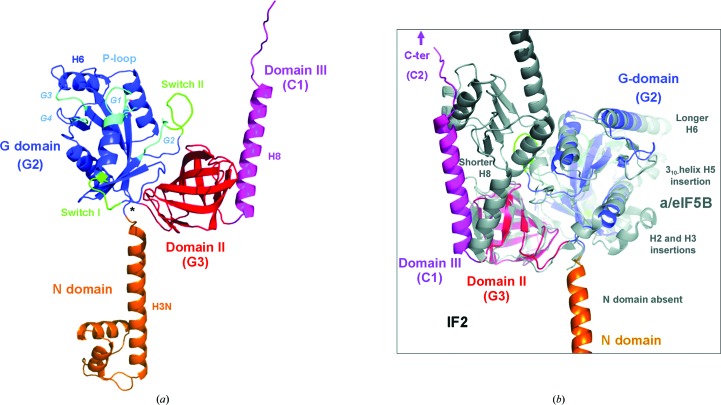

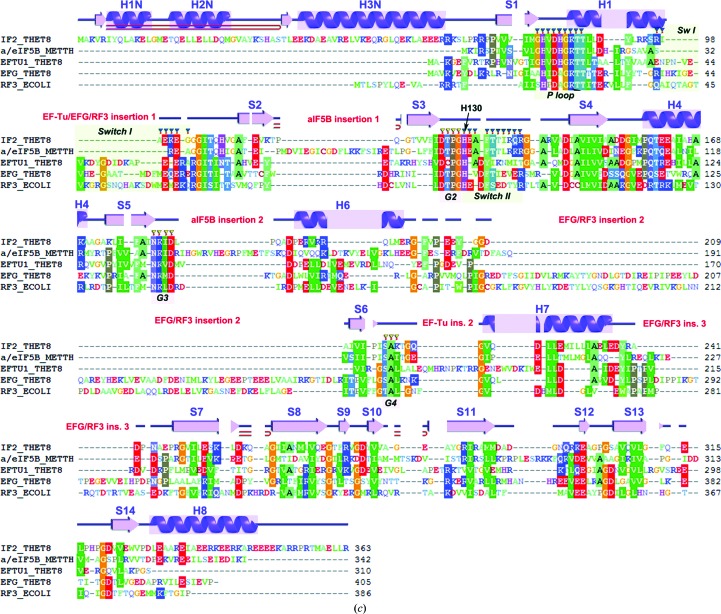
Crystal structure of IF2. (*a*) Overall structure of *T. thermophilus* IF2 (residues 1–363). Domains are annotated and colour-coded: N domain, orange; G domain, blue; domain II, red; beginning of domain III, pink. The switch I and II regions are indicated in green and the nucleotide-binding motifs G1 (P-loop), G2, G3 and G4 are highlighted in cyan. Helix H3N from the N domain and helix H8 from domain III are indicated. (*b*) Comparison of the *T. thermophilus* IF2 (this work) and *M. thermoautotrophicum* a/eIF5B (PDB entry 1g7t, GDPNP complex; Roll-Mecak *et al.*, 2000 ▶) structures showing significant structural differences between the eubacterial and archaeal homologues [IF2 domains are colour-coded as in (*a*); the view focuses on the structurally conserved core comprising the G domain and domain II; the view is rotated 180° compared with (*a*) in order to better illustrate the structural differences; the position of the C-terminal region beyond residue 363 of IF2 is indicated by an arrow]. (*c*) Structure-based sequence alignment of *T. thermophilus* IF2 with related proteins (archaeal a/eIF5B from *M. thermoautotrophicum*, *T. thermophilus* EF-Tu, *T. thermophilus* EF-G and *E. coli* RF3). The G1, G2, G3 and G4 motifs that are in the vicinity of the nucleotide are annotated, as well as the switch I and switch II regions.

Diffraction data for crystals of apo IF2, SeMet apo IF2, IF2–GTP and IF2–GDP were collected using a PILATUS detector at the Swiss Light Source synchrotron, Switzerland, with a typical exposure time of 2 s and an oscillation range of 1° for apo IF2 and SeMet apo IF2 on PXI and with an exposure time of 1 s and an oscillation range of 0.1° for the nucleotide complexes on PXIII. Complete SeMet MAD data for apo IF2 were collected at three wavelengths corresponding to peak (λ = 0.97966 Å), inflection (λ = 0.97984 Å) and remote (λ = 0.97800 Å) points with respect to the selenium absorption edge. All X-ray diffraction data were integrated, processed and scaled using the *XDS* software (Kabsch, 2010 ▶; see Table 1 ▶ for statistics). The *SHELXC*/*D*/*E* programs (Sheldrick, 2008 ▶) as implemented in the *HKL*2*MAP* graphical interface (Pape & Schneider, 2004 ▶) were used to find and refine the selenium sites in the SeMet IF2 MAD data producing the initial phase set. Only nine of the 15 possible Se sites were found. Indeed, gel electrophoresis and MALDI peptide mass fingerprinting analysis of the crystals showed that the crystals comprised amino-acid residues 1–363 (40 kDa) of IF2 (Supplementary Fig. 1), probably as a result of a spontaneous proteolytic event that occurred during crystallization. A proteolysis site around Arg363 has been confirmed by ESI-MS^+^ analysis of IF2 under denaturating conditions after degradation. The electron-density map was readily interpretable and automatic tracing was performed with *Auto-Rickshaw* (Panjikar *et al.*, 2005 ▶), which gave an 81% complete initial model that could be extended to encompass all residues (1–363). Refinement was performed against the high-resolution native data using *phenix.refine* (Afonine *et al.*, 2012 ▶). The graphics program *Coot* (Emsley & Cowtan, 2004 ▶) was used for manual model building, visualization and nucleotide placement. The structures of the nucleotide complexes were determined using the structure of apo IF2 as a starting model in molecular replace­ment. The switch I and II loops required tracing using maps at lower contour levels (and residues 102–104 were not well defined in the three structures). Figures were prepared with *PyMOL* (DeLano, 2002 ▶). Fig. 1 ▶(*c*) was prepared with *BioEdit* (http://www.mbio.ncsu.edu/bioedit/bioedit.html) using a structure-based sequence alignment, and the secondary-structure annotation scheme was added using *Adobe Photoshop*. Data-collection and refinement statistics are given in Table 1 ▶. Structural alignment gives root-mean-square deviations (r.m.s.d.s) of 1.6/0.6, 1.5/0.7 and 1.2/0.4 Å for overall/optimized (after rejection of residues with a high r.m.s.d.) superposition of the full apo/GTP, apo/GDP and GTP/GDP complexes, respectively, and of 1.2/0.5, 1.2/0.6 and 1.1/0.3 Å, respectively, based on the G domains. The coordinates and structure factors have been deposited in the PDB with accession codes 4b3x, 4b48, 4b47 for the apo, GTP and GDP complexes, respectively.

## 3. Results and discussion

### 3.1. Structure determination and overall structure

*T. thermophilus* IF2 yielded well diffracting crystals of the N-terminal core structure (residues 1–363) after proteolysis (or possibly self-cleavage) of the C-terminal part during crystallization [see §[Sec sec2]2 and Supplementary Fig. S1; the C-­terminal region (364–571) is involved in the recognition of the initiator tRNA CCA end region]. The structure of the apoprotein was determined by multi-wavelength anomalous dispersion (MAD) phasing (Hendrickson *et al.*, 1990 ▶) using selenomethionine and was refined to 1.95 Å resolution (see Table 1 for data-collection and refinement statistics ▶; see also Fig. 1 ▶). The IF2–GTP and IF2–GDP structures were obtained by nucleotide soaking and were refined to 2.8 and 2.3 Å resolution, respectively (cocrystallization was also attempted but was unsuccessful and provided very small crystals at best; while it is possible that some additional conformational differences may be limited by the crystal lattice, these are probably different again in the fully functional context when IF2 is bound to the ribosome; moreover, cocrystallization would not have been possible with GTP, which can hydrolyze spontaneously if not used in rapid crystal soaking and cooling). The structure of IF2 comprises three main domains, the N domain, the G domain and domain II, and the first part of domain III (C1; Fig. 1 ▶ *a*). For consistency reasons, we follow the overall ribosomal GTPase-domain nomenclature of EF-Tu (Nissen *et al.*, 1995 ▶) and the secondary-structure numbering of the archaeal *Methanobacterium thermoautotrophicum* a/eIF5B crystal structure (Fig. 1 ▶ *b*; Roll-Mecak *et al.*, 2000 ▶), although *T. thermophilus* IF2 shows several significant differences (see the structure-based sequence alignment in Fig. 1 ▶ *c*). The N domain protrudes from the core formed by the G domain and domain II and is oriented opposite to domain III, which extends on the C-terminal side towards the remainder of the C-terminal region. The IF2-specific N domain (residues 1–69) is composed of two small α-helices (annotated H1N and H2N) folded upon the tip of a 50 Å long α-helix (H3N). The G domain (residues 70–240) comprises a nucleotide-binding fold related to those of ribosomal GTPases (EF-Tu, EF-G, RF3, a/eIF5B *etc.*) and other G proteins (Ras, Ran *etc.*). In the G domain, the sequence conservation is particularly high with regard to four sequence elements (the G1/P loop, G2, G3 and G4; see the structure-based sequence alignment in Fig. 1 ▶ *c*). Helix H1 of the IF2 G domain contains the P-loop region, a loop structure that appears to form by opening up the first helical turn of the α-helix and which harbours the main part of the nucleotide-binding site. The G domain contains two rather flexible loops (switch I and II; residues 97–103 and 130–139, respectively) which adopt different conformations in the apo, GTP-bound and GDP-bound states. Domain II (residues 241–­326) contains an oligonucleotide/oligosaccharide-binding (OB) fold similar to those of the other ribosomal GTPases but with significant sequence differences (Fig. 1 ▶ *c*). The β-barrel is complemented on its side by a long α-helix (H8; Fig. 1 ▶ *a*) which constitutes the beginning of domain III and serves as a linker.

The protein fold resembles that of a/eIF5B to some extent, but with notable differences because a whole series of archaeal sequence insertions are absent in eubacterial IF2 (see Fig. 1 ▶ *c*). For example, helices H2 and H3 in the *M. thermoautotrophicum* a/eIF5B crystal structure (Roll-Mecak *et al.*, 2000 ▶) represent insertions between β-strands S2 and S3, and the region after β-strand S5 that comprises a loop, 3_10_-helix H5 and the beginning of helix H6 in *M. thermoautotrophicum* a/eIF5B is absent in *T. thermophilus* IF2. Moreover, the functionally important switch I region and the P-loop are not visible in the *M. thermoautotrophicum* apo a/eIF5B crystal structure, while the P-loop becomes ordered upon nucleotide binding. The conformation of the P-loop is similar to that observed for IF2. The residue equivalent to His130 (His80) is oriented towards the nucleotide (GDPNP complex), but no water molecule is visible in vicinity of the γ-phosphate. The N domain is absent in archaea, but the fact that it is present in eubacteria and that it folds into a well defined region suggests that it may have a specific function. Based on the IF2 structure, new experiments can be designed to address this question.

### 3.2. Conformational changes of IF2 upon nucleotide binding that control switches I and II in the G domain

**Figure 2:**
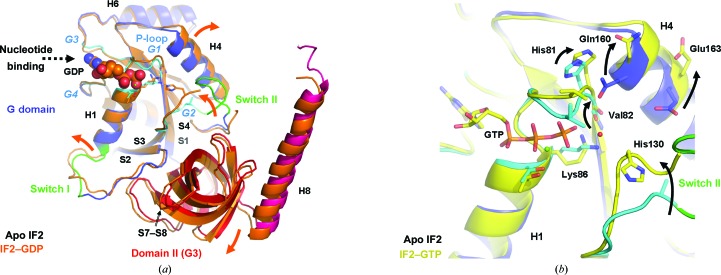
Conformational changes of IF2 upon nucleotide binding. (*a*) The superposition of the apo IF2 (colour-coded as in Fig. 1 ▶ *a*) and the IF2–GDP complex (in orange) shows the overall conformational changes of IF2 that occur upon nucleotide binding (highlighted by arrows; important secondary-structure elements are annotated as in Fig. 1 ▶; for simplicity, the N domain is not shown). (*b*) Detailed view of the nucleotide-dependent conformational changes, revealing the key role of Val82 in the P-loop that transmits conformational changes to Gln160 of helix H4 and the concomitant conformational change of the switch II region (apo IF2 colour-coded as in Fig. 1*a* and IF2–GTP in yellow). The transmission of conformational changes occurs at the cross-over of two loops (marked with an asterisk in Fig. 1 ▶ *a*) that connect the ends of the G domain to the neighbouring domains, suggesting that this region could act as a node coordinating nucleotide-dependent domain movements.

Upon nucleotide binding a strong conformational change occurs in and around the P-loop (Figs. 2 ▶ *a* and 2 ▶ *b*), which opens up to accommodate the β- and γ-phosphate moieties of the nucleotide (when the nucleotide is absent the binding site is filled with water molecules). This involves a large conformational change of Val82, which flips its side chain and moves 2.9 Å towards helix H4. A steric clash with Gln160 of H4 is avoided by pivoting helix H4 7° outwards (Fig. 2 ▶ *a*), a conformational change that is also transmitted to helix H6. The conformational rearrangements around Val82 appear to be functionally relevant because mutation of the corresponding valine residue in *E. coli* (V400G) leads to increased GTP affinity (probably because of fewer steric clashes of a glycine than a valine with the γ-phosphate; Fig. 2 ▶ *b*) but reduced GTP-hydrolysis activity (Luchin *et al.*, 1999 ▶). The conformational cascade (Fig. 2 ▶ *a*) from Val82 to the P-loop and to helices H4/H6 extends to regions in domain II that are part of the 30S binding site in 30S ICs and involve 16S rRNA helices H14 and H5 (Simonetti *et al.*, 2008 ▶). The nucleotide-induced opening of the P-loop leads to a pivoting of the C-terminal end of helix H1 by 10°, which directly affects the conformation of the switch I region (Fig. 2 ▶ *a*). The switch I conformation varies significantly between the apo and nucleotide-bound states, probably owing to the presence of two glycines (RIAEK­EA**GG** sequence) which are highly conserved and provide flexibility (as also illustrated by higher *B* factors). Residues 107/108 located shortly after the switch I loop stabilize this region by an inter-domain contact involving the S11–S12 loop of domain II; in the isolated G domain the switch I region is even more flexible (Wienk *et al.*, 2012 ▶). Switch II adopts rather different conformations in the apo, GTP-bound and GDP-bound states (Fig. 2 ▶ *b*) and is more flexible than the rest of the structure (high *B* factors and only visible in low-contour maps). It is in the vicinity of the P-loop and helix H4 but shows few direct contacts with these (Fig. 2 ▶ *b*), with the exception of the apo IF2 state. In the nucleotide complexes, switch II residues 132–137 are not fully defined, but the catalytically important His130 is defined (it is conserved in EF-Tu, EF-­G and *E. coli* and *Bacillus stearothermophilus* IF2; Fig. 1 ▶ *c*; Cool & Parmeggiani, 1991 ▶; Luchin *et al.*, 1999 ▶; Gualerzi *et al.*, 2001 ▶; Daviter *et al.*, 2003 ▶). The loop region comprising switch II and the G2 motif (Fig. 2 ▶ *a*) is flanked by two strictly conserved glycine residues (sequence **G**HEAFTTIRQR**G**) which confer additional conformational freedom to this region. Hydrogen bonds to β-strands of domain II (Fig. 2 ▶ *a*) help to position the switch II loop (while it adopts a conformation pointing away from the G domain when domain II is missing; Wienk *et al.*, 2012 ▶). The positioning of switch II may have implications in GTP hydrolysis, as discussed below.

### 3.3. Molecular recognition in the GTP-binding site

**Figure 3:**
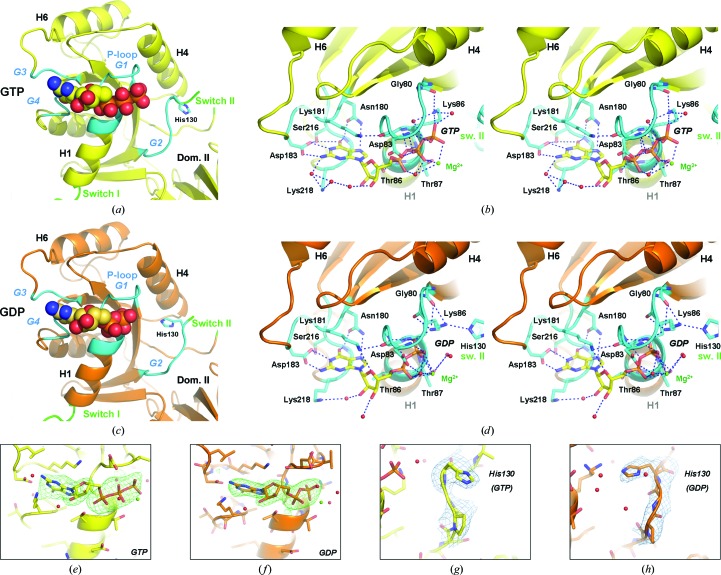
Molecular recognition and GTP/GDP-dependent conformational changes in the catalytic site of IF2. (*a*, *c*) Overall binding site of the nucleotides as observed in the IF2–GTP and IF2–GDP complexes. The nucleotide is inserted in the P-loop (G1 motif) and between the G2, G3 and G4 motifs (in cyan; see sequence alignment in Fig. 1 ▶ *c*); switches I and II are indicated in green. (*b*, *d*) Detailed hydrogen-bond pattern of the IF2–GTP and IF2–GDP complexes (stereo representations). Amino-acid residues interacting *via* their polypeptide backbone or their side chains are indicated; hydrogen bonds are indicated by dotted lines. The guanosine moiety of the nucleotide interacts mostly with the G3 and G4 motifs and the ribose moiety shows hydrogen bonds to Lys218 mediated through water molecules (red spheres), while the phosphate groups interact with residues from the P-loop. Lys181 helps to stabilize the G3 loop with the P-loop. The Mg^2+^ ion (in green) interacts with the β- and γ-phosphate moieties of the GTP, and with the GDP α- and β-­phosphate moieties and with Thr87 in the IF2–GDP complex. The position of the Mg^2+^ ion is almost identical in both complexes, while the β-­phosphate moiety is rotated. An octahedral coordination of the Mg^2+^ ion is not observed, probably because the switch I region is much shorter than in translation elongation factors; additional coordination may potentially be provided by the 50S ribosomal subunit when IF2 is ribosome-bound. Lys86 (P-­loop) interacts with the terminal phosphate group of the nucleotide and changes conformation between the GTP-bound and GDP-bound states, while His130 interacts with Lys86 specifically in the GDP complex. In the GTP complex a water molecule is positioned next to the γ-phosphate. The side chain of His130 is flipped out in the GTP-bound state (not visible in this view) and flipped in in the GDP-bound state. Leu184 is not shown for simplicity (it would overlap with Asp183). (*e*, *f*) OMIT maps of the GTP and GDP complexes shown together with the final refined structures. (*g*, *h*) Electron density of the His130 region for the GTP–IF2 and GDP–IF2 complexes (shown for residues 128–130 for simplicity), providing experimental evidence for the conformational change of His130 and the switch II loop.

The nucleotide-binding site is located in the P-loop region of the G domain and is surface-exposed rather than being a closed ligand pocket. This is in part owing to the fact that the switch I region (14 residues) is much shorter in IF2 than in EF-­Tu (32 amino acids; Nissen *et al.*, 1995 ▶) EF-G (33 amino acids, disordered; Laurberg *et al.*, 2000 ▶) or RF3 (40 amino acids; Gao *et al.*, 2007 ▶). Therefore, in IF2 it cannot reach the nucleotide-binding site, while in the other factors the switch I region forms a secondary structure located next to the nucleotide. The binding site is thus formed solely by the four highly conserved sequence patches in the G domain denoted G1/P, G2 (switch II), G3 and G4 (Figs. 3 ▶ *a* and 3 ▶ *c*), which adopt loop structures and provide the amino acids that are involved in nucleotide recognition and binding. G1/P (residues 80–88) contains a GHVDHGKT(T/S) motif which is formed by the last residue of β-strand S1, the P-loop and the beginning of helix H1. It provides numerous hydrogen bonds to the β- and γ-phosphate moieties of the nucleotide (Figs. 3 ▶ *b*, 3 ▶ *d*, 3 ▶ *e* and 3 ▶ *f*), such as through the main-chain carbonyl group of Gly80 and the side chain of Lys86. G2 contains a DTPGH motif (residues 125–130) which is part of the switch II loop. G2 is in the vicinity of the γ-phosphate moiety, but it does not interact with the nucleotide, with the exception of residue His130 which interacts with Lys86 specifically in the GDP complex (Fig. 3 ▶ *d*; in the GTP complex there is an additional water molecule next to the γ-phosphate moiety; Fig. 3 ▶ *b*). G3 contains an NK(I/M)D motif (residues 180–183) and provides numerous hydrogen bonds to the guanine moiety (Figs. 3 ▶ *b* and 3 ▶ *d*). Leu184 forms a close van der Waals contact with the 2-­amino group of the guanine (3.2–3.4 Å); in most other eubacteria this residue is replaced by Lys or Arg (Fig. 1 ▶ *c*), which could provide additional hydrogen bonding to the nucleotide. G4 contains an (I/V)SAK motif (residues 215–218) in which Lys218 forms water-mediated hydrogen bonds to the ribose (Figs. 3 ▶ *b* and 3 ▶ *d*). Taken together, the sequence conservation of the G1/P, G2, G3 and G4 loops (Fig. 1 ▶ *c*) provides specific recognition of the nucleotide through numerous hydrogen bonds. An adenine moiety in the case of ATP or ADP would not provide the same hydrogen-bond donor/acceptor activities as the 6-­keto and 2-amino groups of the guanine and would therefore reduce the number of hydrogen bonds that contribute to high-affinity binding of the nucleotide.

### 3.4. GTP/GDP-dependent conformational changes in the catalytic site

Comparison of the GTP and GDP complexes of IF2 reveals several significant differences within the nucleotide-binding site (Fig. 3 ▶). The largest conformational changes are observed for residues located around the γ-phosphate group. The Val82 side chain, which moves strongly upon nucleotide binding in order to comply with reasonable van der Waals distances to the γ-phosphate, rotates slightly between the GTP-bound and GDP-bound states. Another important residue is Lys86, which is located right below Val82 in the direct vicinity of the γ-­phosphate group. This residue is conserved in eubacterial IF2s and in ribosomal GTPases (Fig. 1 ▶ *c*). Its flexible side chain changes conformation between the two states: in the presence of GTP (Fig. 3 ▶ *b*) it adopts a bent conformation to form a strong hydrogen bond to the γ-phosphate O atom (2.5 Å). In the presence of GDP (Fig. 3 ▶ *d*) Lys86 has a more extended conformation and is shifted by almost 2 Å into the space left free by the missing γ-phosphate. In all states Lys86 also forms a hydrogen-bond interaction with the carbonyl backbone of Gly80. His130, which is located at the junction of the G2 motif and the switch II loop, seems to be correlated with the conformation of Lys86. In the GTP-bound state (Figs. 3 ▶ *b* and 3 ▶ *g*), His130 is oriented away from Lys86 and the γ-phosphate, but in the presence of GDP its orientation flips and thereby creates a hydrogen bond to Lys86 (3.0 Å) in the extended conformation. Upon GTP hydrolysis and phosphate release Lys86 releases its hydrogen bond to the GTP γ-phosphate and becomes available for interaction with His130. In the GTP complex there is no direct interaction between Lys86 and His130, and the position of His130 is replaced by a water molecule which could play a role in GTP hydrolysis. This water molecule is positioned at the correct distance from the γ-phosphate group (2.6 Å) to initiate GTP hydrolysis and is also hydrogen-bonded to Lys86 (Fig. 3 ▶ *b*). In the GDP complex this water molecule is absent and the hydrogen bonding is replaced by His130 (Figs. 3 ▶ *b* and 3 ▶ *h*). In the apo state the side chain of His130 is not visible in the map, indicating that its role is nucleotide-dependent.

## 4. Conclusions

Taken together, the structures of the IF2–GTP and IF2–GDP complexes presented here suggest an important role of Lys86 (P-loop) and His130 (switch II) in nucleotide binding and/or GTP hydrolysis, as is evident from the proximity of these residues to the phosphate group of the nucleotide (Figs. 3 ▶ *b* and 3 ▶ *d*). Both residues are highly conserved in ribosomal GTPases (Fig. 1 ▶ *c*; they correspond to Lys24 and His85 in *T. thermophilus* EF-Tu, Lys25 and His87 in *T. thermophilus* EF-G and Lys26 and His92 in *E. coli* RF3, respectively). While the corresponding histidine residue is known to be involved in GTP hydrolysis in the case of EF-Tu, EF-G and *E. coli* and *B. stearothermophilus* IF2 (Cool & Parmeggiani, 1991 ▶; Luchin *et al.*, 1999 ▶; Gualerzi *et al.*, 2001 ▶; Daviter *et al.*, 2003 ▶), the importance of the conserved lysine residue highlighted by the structures reported here remains to be analyzed in more detail. However, it is striking that the histidine adopts two nucleotide-dependent conformations. In the presence of GTP His130 is rotated away from the nucleotide, clearly in order to avoid uncontrolled GTP hydrolysis occurring; in the GDP-bound state Lys86 occupies the place of the GTP γ-phosphate and forms a hydrogen bond to His130. It is therefore likely that when activated by the 50S ribosomal subunit in the full ribosome context His130 adopts a conformation similar to that observed in the GDP complex, in which it is perfectly positioned to activate the water molecule located next to the γ-­phosphate. This is consistent with the observation that the conformation of the switch II loop which carries His130 depends on the type of bound nucleotide, suggesting that the switch II positioning has direct implications in GTP hydrolysis and that activation of the 50S-dependent GTPase activity of IF2 occurs through His130 flipping in to become properly positioned for catalysis. Details of the GTP-hydrolysis mechanism will require a deeper functional and structural analysis of 30S and 70S ICs with IF2 bound.

## Supplementary Material

PDB reference: IF2, apo, 4b3x

PDB reference: GTP complex, 4b48

PDB reference: GDP complex, 4b47

Supplementary material file. DOI: 10.1107/S0907444913006422/en5538sup1.pdf

## Figures and Tables

**Table 1:** Data-collection and structure-refinement statistics The SeMet data were used for phasing and initial model building and the apo IF2 structure was refined against the native data; the other data sets were used for the IF2–GTP and IF2–GDP complexes. The varying atom numbers are owing to partial disorder of the switch I and II regions and the beginning of domain III. Values in parentheses are for the highest resolution shell. *R*_merge_ is defined according to *XDS* (Kabsch, 2010 ▶); *R*_work_ and *R*_free_ are crystallographic *R* factors calculated for the work and test data sets (Brünger, 1992 ▶).

	SeMet					
	Peak	Inflection	Remote	Native	IF2–GTP	IF2–GDP
Data collection						
Beamline	PX, SLS			PX, SLS	PXIII, SLS	PXIII, SLS
Space group	*P*2_1_2_1_2_1_			*P*2_1_2_1_2_1_	*P*2_1_2_1_2_1_	*P*2_1_2_1_2_1_
Unit-cell parameters						
*a* (Å)	45.19			45.42	45.02	44.75
*b* (Å)	60.93			61.46	61.95	62.45
*c* (Å)	160.74			162.40	160.42	160.11
Resolution (Å)	50–2.4 (2.5–2.4)	50–2.4 (2.5–2.4)	50–2.4 (2.6–2.5)	50–1.95 (2.05–1.95)	60–2.8 (2.82–2.8)	50–2.3 (2.4–2.3)
*R*_merge_ (%)	7.1 (30.9)	6.5 (28.3)	15.6 (60.4)	9.7 (43.7)	12.1 (88.2)	13.6 (80.2)
No. of reflections	18011	18029	15688	36057	11636	15818
Completeness (%)	99.6 (99.7)	99.5 (99.4)	98.7 (99.6)	100.0 (100.0)	99.9 (100.0)	76.4 (49.8)
Multiplicity	4.7 (4.3)	4.8 (4.5)	5.0 (4.9)	11.0 (11.1)	6.3 (6.2)	3.3 (3.1)
〈*I*/σ(*I*)〉	15.7 (6.1)	16.7 (6.6)	13.9 (4.7)	17.6 (4.2)	15.4 (2.6)	12.4 (2.2)
Refinement						
Resolution limits (Å)				49.0–1.95 (2.0–1.95)	43.3–2.8 (3.1–2.8)	40.6–2.3 (2.4–2.3)
No. of reflections				34052	11629	15810
Protein atoms				2909	2692	2705
Water molecules				204	21	71
*R*_work_/*R*_free_ (%)				17.9/21.9 (21.7/27.0)	22.6/30.4 (32.2/40.6)	21.7/28.6 (30.0/41.3)
Average *B* value for all atoms (Å^2^)				47.7	89.9	70.9
R.m.s. deviations from ideal values						
Bond lengths (Å)				0.013	0.005	0.004
Bond angles (°)				1.38	1.08	0.91
Dihedral angles (°)				16.2	18.5	15.2
Planar groups (Å)				0.007	0.004	0.003
Ramachandran statistics (%)						
Favoured				95.73	95.36	96.54
Allowed				4.00	4.64	3.46
Outliers				0.27	0.00	0.00

## Footnotes

1 Supplementary material has been deposited in the IUCr electronic archive (Reference: EN5538). Services for accessing this material are described at the back of the journal.
