# Supplementary material for: Structure of the protein core of translation initiation factor 2 in apo, GTP-bound and GDP-bound forms
Source: Acta Crystallogr D Biol Crystallogr. 2013 Apr 23;69(Pt 6):925–33. doi: 10.1107/S0907444913006422 (PMC3663118; doi:10.1107/S0907444913006422)
Supplement: Supplementary file 1 [file d-69-00925-sup1.pdf]

Supplementary Material and Figures to

Crystal structure of the protein core of translation initiation factor IF2 in apo, GTP and GDP forms

Angelita Simonetti[1], Stefano Marzi[2], Attilio Fabbretti[3], Isabelle Hazemann[1], Lasse Jenner[1], Alexandre Urzhumtsev[1,4], Claudio O. Gualerzi[3] and Bruno P. Klaholz[1]*

[1] *IGBMC (Institute of Genetics and of Molecular and Cellular Biology), Department of Integrative Structural Biology, Centre National de la Recherche Scientifique (CNRS) UMR 7104 / Institut National de la Santé de la Recherche Médicale (INSERM) U964 / Université de Strasbourg, 1 rue Laurent Fries, 67404 Illkirch, France.*

[2] *Architecture et Réactivité de l'ARN, UPR 9002 CNRS, IBMC (Institute of Molecular and Cellular Biology), 15 rue R. Descartes, 67084 Strasbourg, France; Université Louis Pasteur de Strasbourg, Strasbourg, F-67000 France.*

[3]*Laboratory of Genetics, Department of Biosciences and Biotechnology, University of Camerino, 62032 Camerino (MC), Italy.*

[4]*Université de Lorraine, Physical Department, Vandoeuvre-lès-Nancy, 54506, France.*

To whom correspondence should be addressed. klaholz@igbmc.fr, Tel. ++33 388655755; FAX ++33 388653276

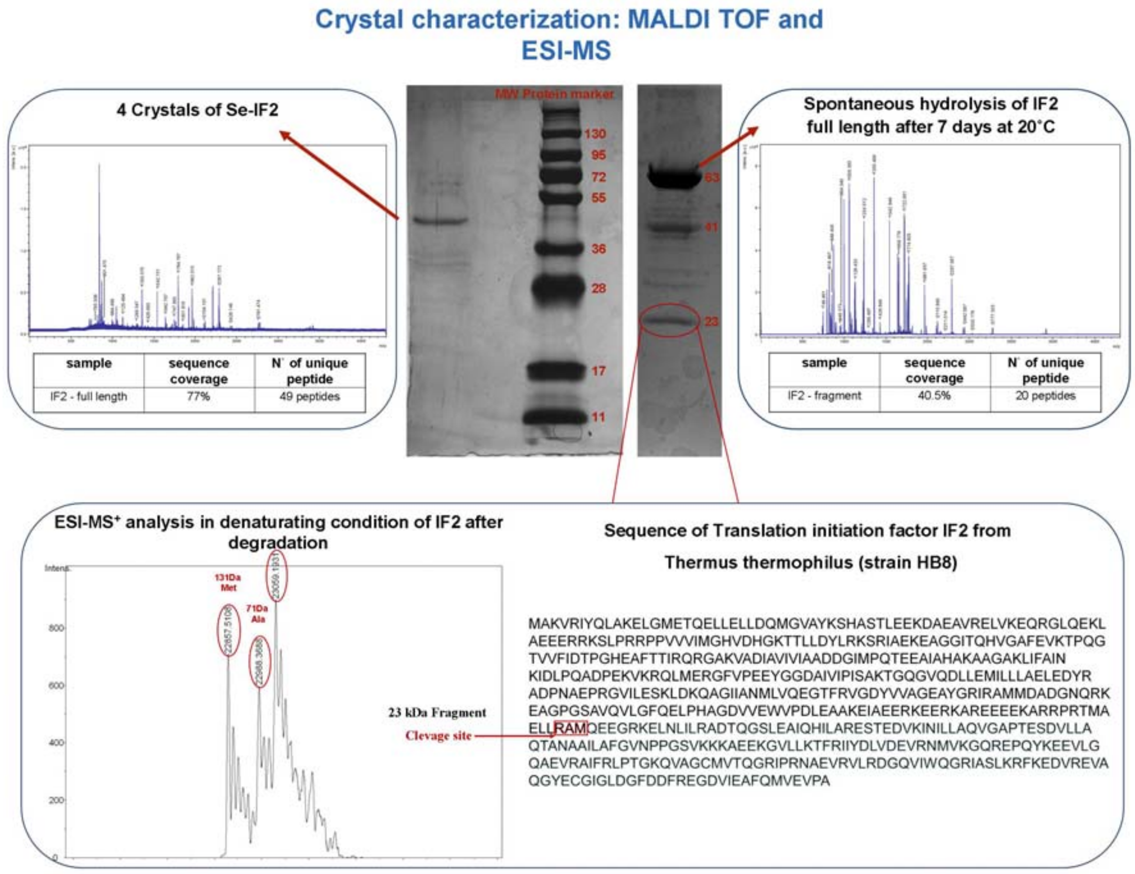

Characterization of IF2

The characterization of crystal content by MALDI-TOF and ESI-MS$^+$ analysis in denaturating conditions of IF2 after degradation revealed that the C-terminal part of IF2 got cleaved off during the crystallization process at position 363 close to a known protease site (residues ~362-365; Szkaradkiewicz, K, Zuleeg, T, Limmer, S, and Sprinzl M., 2000. Interaction of fMet-tRNA^{fMet} and fMet-AMP with the C-terminal domain of *Thermus thermophilus* translation initiation factor 2. *Eur. J. Biochem.* 267, 4290-4299). Proteolysis also occurs in solution and is visible after one week at 4°C. To our knowledge such cleavage is not known for elongation factors.

Suppl. Fig. S1
